# Nurses’ feeling trusted and knowledge hiding: The role of psychological safety, felt obligation and traditionality

**DOI:** 10.3389/fpsyg.2022.1034882

**Published:** 2022-11-18

**Authors:** Guangli Lu, Yipei Liang, Yueming Ding, Haishan Tang, Yiming Zhang, Haitao Huang, Chaoran Chen

**Affiliations:** ^1^Institute of Business Administration, School of Business, Henan University, Kaifeng, China; ^2^Institute of Nursing and Health, School of Nursing and Health, Henan University, Kaifeng, China

**Keywords:** feeling trusted, psychological safety, felt obligation, knowledge hiding, traditionality

## Abstract

Knowledge hiding is one of the dilemmas of organizational knowledge management. For nurses, knowledge hiding behavior is not conducive to improving the quality and efficiency of their work and hinders the innovation of nursing services. Based on the social exchange theory, the current study constructed a moderated mediation model by taking psychological safety and felt obligation as mediating variables, and traditionality as moderating variable, and explored the mechanism of feeling trusted affecting knowledge hiding behavior. The empirical research based on 285 nurses from China shows that feeling trusted is negative correlate with knowledge hiding behavior; feeling trusted can negatively affect knowledge hiding by enhancing psychological safety and felt obligation; traditionality can positively moderate the relationship between feeling trusted and felt obligation, and feeling trusted has a stronger positive influence on felt obligation of highly traditional nurses; traditionality has no significant moderating effect between feeling trusted and psychological safety. Theoretically, this study supplements the influencing factors of knowledge hiding, examines the complex mechanism between feeling trusted and knowledge hiding and supplements the boundary conditions for feeling trusted to play its role from the perspective of individual characteristics (i.e., traditionality). From the perspective of practical implication, this study suggests that managers should pay attention to using trust strategies to enhance subordinates’ psychological safety and felt obligation, especially for highly traditional nurses, thus reducing knowledge hiding.

## Introduction

In the era of knowledge economy, knowledge has gradually become one of the most direct and important production factors in organizations (Koay and Lim, 2021; Ma and Zhang, 2021).With the rapid changes of the external society and environment, organizations need constant innovation to gain and maintain competitive advantage (Qi and Ramayah, 2022), which largely relies on effective knowledge management (Alavi and Leidner, 2001; Wang and Dong, 2022). Knowledge management cannot be separated from people and technology (Becerra-Fernandez, 2000; Pai et al., 2022). As far as technology is concerned, the development and application of modern information technologies such as big data, cloud computing, blockchain and the Internet of Things have improved the efficiency of knowledge management (Abbate et al., 2022). However, as the carrier and transmitter of knowledge, if people do not give full play to their initiative and flexibility to spread, share and apply knowledge, the effect of knowledge management will be greatly affected (Currie and Kerrin, 2003). At present, a kind of headache for managers is widespread in organizations, that is, knowledge hiding (Peng, 2013; Singh, 2019). Studies have shown that employees in smart healthcare firms, telecom sector, the government sector and academic institutions all reported that they had the experience of deliberately hiding knowledge (Cao, 2022; He and Wei, 2022; Qi and Ramayah, 2022; Wang and Dong, 2022).

Knowledge hiding refers to the behavior of deliberately hiding knowledge from others by evading, playing dumb and providing reasonable reasons when faced with other people’s knowledge requests (Connelly et al., 2012). This kind of subjective and intentional behavior that hinders knowledge transfer may bring great resistance to the development of the organization. It is found that knowledge hiding will destroy interpersonal trust, cause tension among employees, and is not conducive to the unity and harmony of the organization (Cerne et al., 2014). It increases the pressure to maintain relationships, leading to unnecessary energy loss and affects job performance (Jahanzeb et al., 2020). In addition, knowledge hiding will affect knowledge transfer in the organization and increase the cost of searching and acquiring knowledge, which not only affects work efficiency, but also hinders organizational innovation (Fong et al., 2018).

At present, there is little research on the knowledge hiding behavior of nurses. Although some scholars think that nurse is a profession with high professional commitment, and may not refuse other people’s knowledge requests (Connelly et al., 2012), in fact, knowledge hiding also exists among nurses. For example, previous studies have found that negative affective states, workplace bullying and intragroup competition will prompt nurses to show knowledge hiding behavior (Zhao and Xia, 2019; Fatima et al., 2021; Sun et al., 2022). It is worth noting that knowledge hiding behaviors of nurses not only have a negative impact on individuals and organizations like other industries, but also may affect the interests of patients (Song et al., 2021).Therefore, it is necessary for us to analyze and discuss the phenomenon of nurses’ knowledge hiding and to explore its influencing factors.

Trust is an important predictor of knowledge hiding behavior (Connelly et al., 2012). Previous studies have found that interpersonal trust, trust in organizations and trust in leaders can negatively predict knowledge hiding (Liu, 2015; Yao et al., 2020; Erkutlu and Chafra, 2021). These studies are all about how an individual’s trust in others affects his/her knowledge hiding behavior from the perspective of trustor. However, how feeling trusted affects knowledge hiding behavior has yet to be explored. Feeling trusted is different from trust. It refers to the trustee’s perception of whether he or she is trusted by others (Lau et al., 2007), including felt reliance and felt disclosure (Lau et al., 2014). The former refers to the degree to which subordinates perceive that their superiors are willing to rely on their work-related knowledge, skills, judgments and behaviors. The latter refers to the degree to which subordinates perceive that their superiors are willing to share sensitive work and personal key information with them (Chen H. et al., 2021). Scholars believe that the trust signal transmitted by the trustors can only play a role if it is received by the trustees so it is necessary to study the relationship between feeling trusted by supervisors and knowledge hiding from the perspective of the trusted person (Baer et al., 2015). Social Exchange Theory is the most dependent theoretical framework to explain the role of trust in motivating employees’ attitudes and behaviors (Fulmer and Gelfand, 2012; Skiba and Wildman, 2019). When subordinates feel the trust from their superiors, they also feel the expectations of their superiors for reciprocity. In this case, in order to maintain the exchange relationship, they may show fewer behaviors that are not in line with the expectations of their superiors. Therefore, based on the social exchange theory, this paper discusses the influence of feeling trusted by superiors on nurse’s knowledge hiding from the perspective of the trusted person (nurse).

In addition, we also explore the mediating mechanism of feeling trusted affecting knowledge hiding. From the motivation of knowledge hiding, we propose a dual mediation mechanism of psychological safety and felt obligation. On the one hand, for employees, knowledge and experience in work are valuable resources, intangible assets and even their competitiveness. Therefore, when employees feel uncertain about the exchange relationship between supervisor and them, they may be reluctant to share knowledge to help organization develop and may hide knowledge for the motivation of protecting knowledge resources and maintaining competitive advantage (Anand and Walsh, 2016; Anand et al., 2019; Han et al., 2021). The trust of superiors will create a safe and reliable organizational atmosphere and meet the psychological safety needs of employees (Ma and Wang, 2014; Song et al., 2020), which will reduce employees’ risk expectation of the exchange process and help to reduce knowledge hiding behavior (He et al., 2020). Based on this, this paper puts forward the mediate role of psychological safety. On the other hand, knowledge hiding may also be due to employees’ cognition that they have no obligation to share. The acquisition and accumulation of knowledge requires a certain amount of time and effort. These investments may make employees have a kind of psychological ownership of their own knowledge (Peng, 2013), thinking that “it is my knowledge and I have no obligation to tell others.” According to the reciprocity norm of social exchange theory, feeling trusted by superiors will increase employees’ willingness and sense of obligation to reciprocate organization (Skiba and Wildman, 2019), so that they may reduce knowledge hiding behavior which is not conducive to organizational development. In view of this, this paper puts forward the mediating role of felt obligation.

Besides, under the background of Chinese culture, due to the differences of individual traditional values, the perception of being trusted by superiors may have different influences on subordinates (Zhang, 2017). Traditionality reflects an individual’s cognition and acceptance of social traditional culture (Farh et al., 2007). There are obvious differences in values and behavior patterns among individuals with different traditionality (Farh et al., 2007). Previous studies have shown that traditionality often plays a moderating role in the influence of leadership style on employees’ behaviors (Liu et al., 2013; Li et al., 2014). However, few studies have paid attention to the role of traditionality in the influence of feeling trusted on employees’ behavior. Therefore, this paper chooses traditionality as a moderating variable to explore whether it has a moderating effect on the relationship between feeling trusted, psychological safety and felt obligation.

To sum up, this paper will discuss the influence of feeling trusted on nurses’ knowledge hiding behavior, and tests the mediating role of psychological safety and felt obligation, as well as the moderating role of traditionality. Our efforts will make four contributions. First of all, how feeling trusted affects knowledge hiding has not been empirically tested. This study will help to understand the influence and mechanism of feeling trusted on knowledge hiding from the perspective of the trustee. Secondly, analyzing from the two mediating paths of psychological safety and felt obligation, this study can explain in detail the complex psychological mechanism of the impact of feeling trusted on knowledge hiding. Thirdly, it is a useful supplement to the boundary conditions of feeling trusted to test the moderating effect of traditionality on the relationship between feeling trusted and psychological safety and felt obligation. Finally, our research conclusion will provide reasonable suggestions for reducing nurses’ knowledge hiding behavior, and provide reference for improving nursing quality and medical service efficiency.

## Theoretical background and hypotheses

In this study, social exchange theory is used to explain the research framework. According to the social exchange theory, the social exchange relationship is based on “paid” exchange, and the parties will engage in and maintain the exchange relationship with others under the expectation of getting a return from others (Homans, 1958; Blau, 1964).

There is uncertainty in social exchange relationships, mainly because it is unclear whether the other party will repay their contributions. Before the establishment of the exchange relationship, individuals will evaluate the possible uncertainties and risks in the exchange process (Blau, 1964). The result of risk assessment will directly affect people’s attitudes and behaviors towards exchange relationships (Molm et al., 2000). Therefore, trust is usually regarded as the basis and starting point of establishing social exchange relationships (Blau, 1964). When one party trusts the other party, or feels the other party’s trust in itself, this uncertainty and risk expectation will undoubtedly decrease. For employees, the degree of risk assessed in the process of establishing social exchange relationships with their superiors or organizations can be expressed as their psychological safety (Su and Liu, 2018). When employees’ evaluation of risks is low, it means that employees feel higher psychological safety perception in the organization, which is conducive to forming an exchange relationship with superiors or organizations (He et al., 2020).

In addition, reciprocity is the core of social exchange. Both parties involved in social exchange not only hope to get due compensation and reward in the exchange relationship, but also have the responsibility and obligation to compensate and repay others. Gouldner (1960) believes that in the process of social exchange, people would take the initiative to do something to repay those who helped and supported themselves, or not to do something that might harm the interests of benefactors. If one party benefits in the process of social exchange without making a return to the other party, it will violate the principle of reciprocity in the process of social exchange, and the social exchange behavior will be terminated. Therefore, in the social exchange relationship, the party who gets the benefits provided by others is bound by the reciprocity norm, and will have a sense of “debt” and obligation to others, thus actively repaying the other party to maintain this exchange relationship.

In this study, based on the social exchange theory, we set up a dual-path model from feeling trusted to knowledge hiding. On the one hand, feeling trust satisfies nurses’ psychological safety perception. In this case, they may be willing to establish exchange relationships with superiors or organizations and make behaviors beneficial to the organization as a response. On the other hand, feeling trusted arouses nurses’ felt obligation to the organization and they need to do something beneficial to the organization in return. Both paths will negatively affect knowledge hiding.

### Feeling trusted and knowledge hiding

According to the social exchange theory, the two parties in the exchange relationship will follow the principle of fairness and reciprocity to exchange, otherwise, the exchange relationship will not last (Blau, 1964). The trust of superiors is a gift for employees (Skinner et al., 2014) and feeling trusted by superiors just like getting a “gift.” In order to maintain this exchange relationship, employees who feeling trusted by superiors need to do some behaviors in line with the expectations of their leaders in return (Kierein and Gold, 2000). Previous studies have found that feeling trusted will encourage employees to exhibit more constructive behaviors toward the organization, such as proactive behavior, voice behavior and organizational citizenship behavior (Lau et al., 2014; Yu, 2019; Chen H. et al., 2021). Similarly, employees may repay their superiors’ trust by reducing negative behaviors, such as reducing silence behavior (Zhu et al., 2019), as well as knowledge hiding. Knowledge hiding behavior is generally regarded as self-interested behavior that ignores organizational development in order to maintain one’s own advantages (Jiang et al., 2019). However, when employees perceive the trust from their superiors, they will have a sense of responsibility to safeguard the interests of leaders (that is, the interests of the organization; Chen X. et al., 2021), and may be willing to accept the knowledge request from others, thus reduce knowledge hiding. Therefore, we speculate that the stronger the nurses’ feeling trusted, the less the nurses’ knowledge hiding behavior.

*H1*: Feeling trusted is negatively related to nurse’s knowledge hiding behavior.

### The mediating role of psychological safety

According to the social exchange theory, whether employees are willing to establish social exchange relationships with organizations through reciprocal behaviors depends on employees’ identification and evaluation of exchange risks (Molm et al., 2000). If employees think that the risk of establishing an exchange relationship with the organization is low, then they will have a willingness to exchange and further implement it into action (Liu and Wang, 2022). Psychological safety level represents this risk assessment result to a certain extent (Su and Liu, 2018). Psychological safety refers to the psychological perception that an individual can show their true self in the organization without worrying that their image and development will be destroyed (Kahn, 1990). It describes the employees’ perception that they feel at ease about the risk of their behavior in the organization (Edmondson, 1999). Psychological safety relies on high levels of interpersonal support, trust, respect, and fellowship (Kahn, 1990; He et al., 2020). For employees, the superiors are the agent or representative of the organization. The evaluation of the superiors often represents the attitude and will of the organization and affects employees’ perception of their working environment (Song et al., 2020). Therefore, when nurses feel that their superiors trust themselves, they undoubtedly get a safe signal, that is, their organization supports and trusts them, which will meet their psychological safety needs (Ma and Wang, 2014). In this case, employees may be willing to establish and maintain exchange relationships with their superiors and organizations by behaviors that meet their positive expectations (Su and Liu, 2018). Obviously, knowledge hiding does not meet the expectations of superiors, and it is not conducive to the development of the organization (Connelly et al., 2012). Therefore, when psychological safety is satisfied, employees may will to reduce knowledge hiding behavior (He et al., 2020; Men et al., 2020). And psychological safety may be may be the bridge between being trusted and knowledge hiding.

*H2*: Psychological safety will play a negative mediating role between feeling trusted and knowledge hiding, that is, feeling trusted will negatively affect knowledge hiding by enhancing psychological safety.

### The mediating role of felt obligation

The key to operationalizing the effect of trust within the social exchange framework is through feelings of obligation (Colquitt et al., 2012; Skiba and Wildman, 2019). Felt obligation refers to employees’ awareness that they have an obligation to care for the welfare of the organization and help it achieve its goals (Eisenberger et al., 2001). It shows individual perception of their responsibilities in the organization (Wang and Huang, 2019). The social exchange theory points out that both parties in the social exchange relationship will be bound by the principle of reciprocity, and the party who gains benefits needs to pay accordingly, so the exchange relationship will be strengthened and maintained. In the organization context, trust from superiors is often accompanied by psychological and resource support (Yu, 2019). Therefore, feeling the trust from superior and obtaining these benefits will make the trusted person have a psychological feeling of “gratitude and obligation to repay the supervisor” and have the motivation to make efforts to fulfill these obligations as a means to maintain social exchange relationships (Wang and Lv, 2018). The higher the level of felt obligation, the stronger the motivation of employees to help achieve organizational goals. Under the influence of this awareness of the need to fulfill obligations, when faced with other people’s knowledge request, employees may make behavior choices beneficial to the organization and reduce their knowledge hiding behavior. Therefore, nurses’ feeling trusted may be first transformed into a clear cognition, that is, the perception that they need to fulfill their obligations. Then, under the influence of this sense of obligation, knowledge hiding behavior is suppressed.

*H3*: Felt obligation will play a negative mediating role between feeling trusted and knowledge hiding, that is, feeling trusted will negatively affect knowledge hiding by enhancing felt obligation.

### The moderating role of traditionality

Although people living in the contemporary era are constantly impacted by modern culture, traditional values are still branded in Chinese people’s minds and play an important role. Traditionality refers to an individual’s recognition of traditional values and behavior norms (Yang et al., 1989). Zhang (2017) pointed out that faced with the same external stimuli (such as feeling trusted), individuals with different traditionality levels may have different reactions. People with high traditionality have a strong sense of hierarchy and are sensitive to the differences between their superiors and their own status and rights. They have a clear understanding of their position in the organization and abide by the organizational norms, and keep a respectful and obedient attitude towards their superiors and their decisions (Li et al., 2014). However, people with low traditionality have a weak sense of hierarchy, follow the principle of fairness, and tend to think that they have an equal relationship with their superiors and have low compliance with their superiors (Liu et al., 2013).

It can be inferred that individuals with high traditionality are influenced by the idea of being superior and inferior, and they pay more attention to the feedback given by their superiors and react more strongly. When feeling trusted by superiors, high-traditional employees may have a more obvious perception of support and recognition they obtain, thus generating a stronger psychological safety. On the contrary, for employees with low traditionality, the stimulation of feeling trusted by superiors may be weak (Liu et al., 2018). Similarly, when high-traditional employees perceive the trust of their superiors, they may be inspired and have a stronger sense of obligation and responsibility, and be clearer about their own responsibilities in the organization. While the role of feeling trusted on low traditionality employees’ obligation may be relatively weak.

*H4*: Traditionality will moderate the relationship between nurses’ feeling trusted and psychological safety and for high traditionality nurse, the relationship will be stronger.

*H5*: Traditionality will moderate the relationship between nurses’ feeling trusted and felt obligation and for high traditionality nurse, the relationship will be stronger.

**Figure 1:**
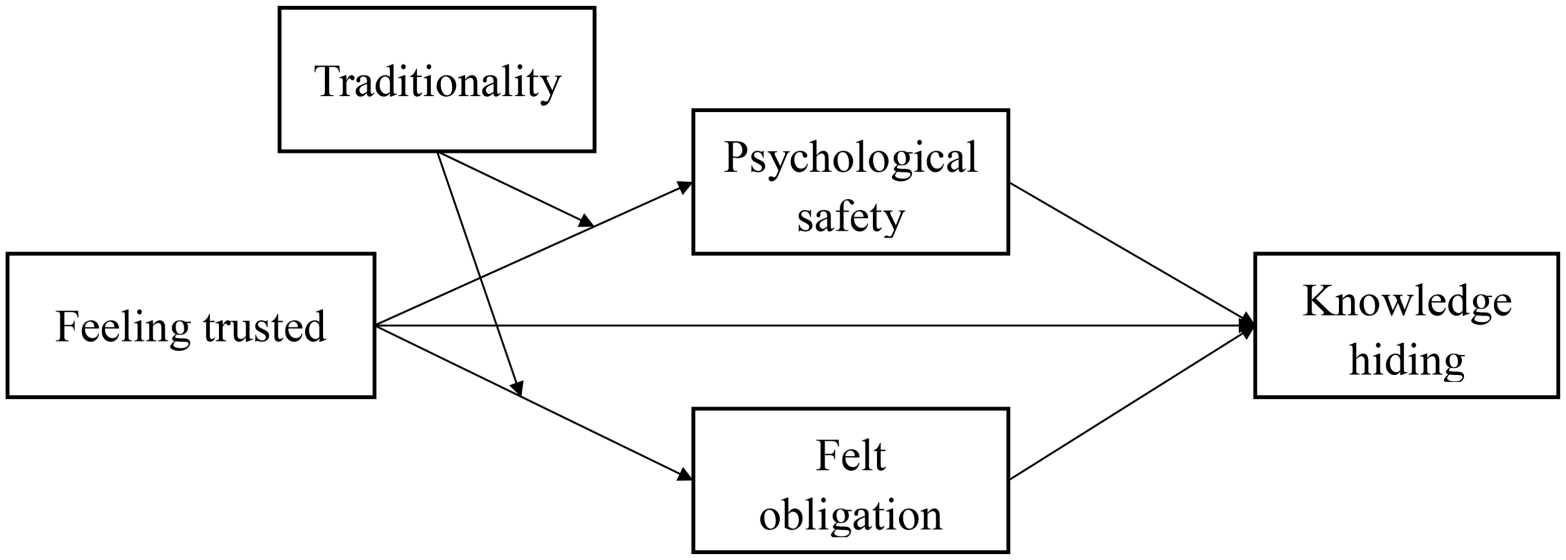
Hypothetical model.

The hypothetical model is shown in Figure 1.

## Materials and methods

### Data collection

#### Sample size calculation

The primary variables in this study are continuous data. Therefore, the method of Barlett et al. (2001) is used to calculate the minimum sample size, as follows:


$$n=\frac{t^{2}\times s^{2}}{d^{2}}=\frac{1.96^{2}\times 1.25^{2}}{{\left(5\times 0.03\right)}^{2}}=266$$

t = value for selected alpha level of 0.05 in two tail = 1.96.

s = estimate of standard deviation in the population = $\frac{\;\mathrm{number of points}\;\mathrm{on}\;\mathrm{the scale}}{\mathrm{number of standard deviations}}=\frac{5}{4}$ = 1.25.

d = acceptable margin of error for mean being estimated = number of points on primary scale × acceptable margin of error = 0.15. (points on primary scale = 5; acceptable margin of error = 0.03).

Therefore, the minimum sample size of this research should be 266.

#### Survey methods

Data were collected by questionnaire survey. Since all variables involved in this study belong to individual psychological perception variables, data measurement adopts self-report. In order to avoid common variance, this study collected data in two time nodes. In the two-wave questionnaires, respondents were asked to fill in the last four digits of their mobile phone numbers to match the research data obtained from the phased investigation. In China, the last four digits of a mobile phone number are user numbers, which are usually used for identity confirmation (Yu X. et al., 2022). It plays a role in identifying and matching identities under the condition of anonymity (Niu and Liu, 2021).

#### Survey procedures

From January 2022 to March 2022, a two-wave questionnaire survey was conducted among nurses in three hospitals in China. In time 1, participating nurses need to fill in basic personal information and complete the measurement of variables such as feeling trusted, psychological safety, felt obligation and traditionality. 400 questionnaires were distributed and 356 were recovered, with a recovery rate of 89%. In Time 2, questionnaires were distributed to 356 nurses who had answered the first questionnaire to measure knowledge hiding variable and 313 of them were recovered. Excluding 19 questionnaires with missing values and 9 questionnaires with obvious regular answers, 285 valid questionnaires were finally obtained, with an effective recovery rate of 80%.

#### Sample characteristics

Among 285 nurses, 25 are male (8.80%), 260 are female (91.20%). In terms of age, 156 are under 30 years old (54.73%), 97 are between 30 and 39 years old (34.04%), 29 are between 40 and 49 years old (10.18%) and 3 nurses are over 50 years old (1.05%). With regard to education level, 54 nurses have junior college or below degree (18.95%), 211 nurses have a bachelor’s degree (74.04%), 20 nurses have a master’s or above degree (7.01%). In terms of organizational tenure, 150 are less than 5 years (52.63%), 66 nurses are between 5 and 10 years (23.16%), 61 are between 10 and 15 years (21.40%) and 8 nurses’ organizational tenure is more than 15 years (2.81%).

### Measures

The scales used in this study have been translated by professionals, and have been well applied in China. All items were measured with a 5-point Likert scale, from 1 to 5 indicates “complete disagreement” to “complete agreement.”

#### Feeling trusted

The scale developed by Gillespie (2003) and revised by Lau et al. (2014) was used to measure feeling trusted, including two dimensions: felt reliance and felt disclosure. The scale consisted of 10 items. An example item was “My supervisor is willing to put me in charge of some important projects.” In this study, Cronbach’s α coefficient of this scale is 0.87. The Cronbach’s α coefficient of the two sub-dimensions are 0.74 and 0.82, respectively.

#### Psychological safety

Psychological safety was measured by a maturity scale developed by Liang et al. (2012), with 5 items in total. A sample item was “I’m not worried that expressing my true thoughts at work will be detrimental to myself.” The higher the score, the higher the level of psychological safety. In this study, Cronbach’s α coefficient is 0.86.

#### Felt obligation

Felt obligation was assessed with a 7-item scale developed by Eisenberger et al. (2001). An example item was “My duty is to try my best to help the organization achieve its goals.” Drawing lessons from previous scholars’ practices, we deleted the inverse question in the questionnaire and the remaining 6 items with high factor load were used to evaluate the felt obligation (Duan et al., 2021). Cronbach’s α coefficient for the scale in this study was 0.82.

#### Traditionality

We measured nurses’ traditionality using a 5-item scale developed by Farh et al. (1997). A sample item is “In order to avoid mistakes, the best way is to listen to seniors.” The scale is the most widely used traditionality measurement scale in the field of organizational management. Cronbach’s α coefficient was 0.68. Farh et al. (2007) pointed out that the low internal consistency coefficient of the scale is due to covering a wide range of fields such as politics, family and social relations.

#### Knowledge hiding

This study adopts the 12-item scale developed by Connelly et al. (2012) to measure knowledge hiding, which includes three dimensions: evasive hiding, playing dumb, and rationalized hiding, with four items each. A sample item is “When faced with requests from others, I will play dumb to avoid knowledge sharing.” Cronbach’s α coefficients of three sub-dimensions in this study are 0.89, 0.83 and 0.88, respectively. Overall Cronbach’s α coefficient of the scale is 0.94.

#### Control variables

Previous studies have shown that population background variables will influence knowledge hiding behavior (Rhee and Choi, 2017; Peng et al., 2021). Therefore, nurses’ gender, age, education and organizational tenure are taken as control variables.

### Ethical consideration

The research was approved by the hospital leaders and Henan University. All participating nurses were informed of the purpose of this research and their right to participate and withdraw at any time during the research period. In addition, we have informed participants that this research is anonymous and promised that their answers will only be used for this research. This study has obtained the informed consent of participants.

### Data analysis

SPSS 26.0 and Mplus 8.3 software were used for data analysis. First of all, Harman’s single-factor test was used to test the common method biases. Secondly, confirmatory factor analysis was used to test the validity of discrimination between variables. Thirdly, descriptive statistics (mean, standard deviation) and correlation analysis are carried out on the variables in the study. Finally, this study used the PROCESS program developed by Hayes (2013) to test the hypotheses. Model 4 was used to test the mediating effect of psychological safety and felt obligation between feeling trusted and knowledge hiding, and model 7 was used to test the moderating effect of traditionality.

## Result

### Common method biases test

We adopted Harman’s single-factor method to test the homogeneity of the data. The result shows that the variance explanation percentage of the first factor is 29.46%, less than 40%. It can be concluded that there are no serious common method biases in this research (Zhou and Long, 2004).

### Confirmatory factor analysis

The research shows that when the sample sizes are smaller than 500, commonly used cutoffs of 0.90 for TLI and CFI tend to over reject acceptable models (Hu and Bentler, 1995; Ma et al., 2011). According to Elsbach and Bhattacharya (2001), acceptable model fitness should be between 0.80 and 0.90 for research with a sample size of less than 500. The sample size in this study is 285, which is applicable to this standard. As shown in Table 1, the fitting index of the five-factor model has reached the standard of “acceptable” or “good,” and it is significantly better than that of the other four alternative models (χ^2^[655] = 1393.341, TLI = 0.857, CFI = 0.867, RMSEA = 0.063; Hu and Bentler, 1995; Elsbach and Bhattacharya, 2001; Ma et al., 2011; Duan, 2012). Therefore, the five variables have good discrimination validity and are five different constructs.

**Table 1:** Confirmatory factor analysis (CFA) of discrimination validity.

Factors	X^2^	df	X^2^/df	CFI	TLI	RMSEA
5factors: FT; PS; FO; T; KH	1393.341	655	2.127	0.867	0.857	0.063
4factors: FT; PS + FO; T; KH	1738.686	659	2.638	0.806	0.793	0.076
3factors: FT + PS + FO; T; KH	1986.303	662	3.000	0.762	0.747	0.084
2factors: FT + PS + FO + T; KH	1989.883	664	2.997	0.761	0.747	0.084
1factor: FT + PS + FO + T + KH	3322.441	665	4.996	0.522	0.495	0.118

*N* = 285. FT = Feeling trusted; PS = Psychological safety; FO = Felt obligation; T = Traditionality; KH = Knowledge hiding.

### Descriptive statistics and correlations

Table 2 shows the mean, standard deviation and Pearson correlation coefficient of variables. Feeling trusted is positively correlated with psychological safety (*r* = 0.634, *p* < 0.01) and felt obligation (*r* = 0.415, *p* < 0.01), but negatively correlated with knowledge hiding (*r* = −0.275, *p* < 0.01). Psychological safety is positively related to felt obligation (*r* = 0.456, *p* < 0.01), but negatively related to knowledge hiding (*r* = −0.359, *p* < 0.01). Felt obligation is negatively related to knowledge hiding (*r* = −0.382, *p* < 0.01). Traditionality has no significant correlation with these variables (*p* > 0.05).

**Table 2:** Descriptive statistics and correlations.

Variables	M	SD	Gender	Age	Education	Tenure	FT	PS	FO	T	KH
Gender	1.91	0.28	1								
Age	1.58	0.72	0.024	1							
Education	1.88	0.50	−0.025	−0.113	1						
Tenure	2.49	1.16	0.079	0.753^**^	−0.050	1					
FT	3.34	0.71	0.130^*^	0.171^**^	−0.102	0.193^**^	1				
PS	3.50	0.81	0.104	0.084	−0.099	0.118^*^	0.634^**^	1			
FO	3.84	0.70	0.072	0.064	0.012	0.149^*^	0.415^**^	0.456^**^	1		
T	2.86	0.59	−0.176^**^	0.028	0.104	0.055	0.067	0.032	0.015	1	
KH	2.10	0.90	−0.079	−0.105	0.034	−0.181^**^	−0.275^**^	−0.359^**^	−0.382^**^	0.097	1

*N* = 285. FT = Feeling trusted; PS = Psychological safety; FO = Felt obligation; T = Traditionality; KH = Knowledge hiding. *p < 0.05; **p < 0.01.

### Main effect and mediating effect test

The results are shown in Table 3. (1) Feeling trusted negatively affects knowledge hiding (Model 5, *β* = −0.247, *p* < 0.001), and Hypothesis 1 is supported. (2) Feeling trusted positively predicts psychological safety (Model 1, *β* = 0.630, *p* < 0.001). As shown in Model 6, after the mediator (psychological safety) is added, psychological safety significantly negatively predicts knowledge hiding (*β* = −0.306, *p* < 0.001), indicating that the mediating effect of psychological safety is established, and hypothesis 2 is supported. At this time, the negative effect of feeling trusted on knowledge hiding behavior decreases and becomes not significant (*β* = −0.054, *p* = 0.456), which indicates that psychological safety plays a full mediating role between feeling trusted and knowledge hiding. (3) Feeling trusted positively predicts felt obligation (Model 3, *β* = 0.408, *p* < 0.001). As shown in model 7, after the mediator (felt obligation) is added, felt obligation significantly negatively predicts knowledge hiding (*β* = −0.312, *p* < 0.001), indicating that the mediating effect of felt obligation is established, and hypothesis 3 is supported. In this case, the negative effect of feeling trusted on knowledge hiding decreases and becomes not significant (*β* = −0.120, *p* = 0.051), which indicates that felt obligation also plays a full mediating role between feeling trusted and knowledge hiding.

**Table 3:** The model of mediating effect with moderating.

Variables	Psychological safety		Felt obligation		Knowledge hiding		
	Model 1	Model 2	Model 3	Model 4	Model 5	Model 6	Model 7
Gender	0.020	0.082	0.010	−0.003	−0.034	−0.028	−0.031
Age	−0.052	−0.080	−0.128	−0.168	0.086	0.070	0.046
Education	−0.039	−0.060	0.047	0.074	0.008	−0.004	0.023
Tenure	0.032	0.022	0.167*	0.156*	−0.195*	−0.185*	−0.142
FT	0.630***	0.628***	0.408***	0.414***	−0.247***	−0.054	−0.120
PS						−0.306***	
FO							−0.312***
T		−0.011		−0.011			
FT*T		−0.086		0.131*			
R^2^	0.405	0.412	0.188	0.206	0.0973	0.153	0.176
F	37.927***	27.750***	12.885***	10.248***	6.016***	8.379***	9.920***

N = 285. FT = Feeling trusted; PS = Psychological safety; FO = Felt obligation; T = Traditionality; KH = Knowledge hiding. *p < 0.05; ***p < 0.001.

Table 4 shows the total effect, direct effect and indirect effect. It can be seen that the total effect of feeling trusted on knowledge hiding is significant (confidence interval is [−0.392, −0.147], excluding 0), feeling trusted has a significant indirect impact on knowledge hiding through psychological safety and felt obligation (confidence intervals are [−0.318, −0.042], [−0.256,-0.044], excluding 0), but the direct effect of feeling trusted on knowledge hiding is not significant (confidence interval is [−0.216, 0.215], including 0). In addition, considering that there are two parallel mediation paths in this study, we compare the differences between the two mediation effects. The result shows that the mediating effect of psychological safety between feeling trusted and knowledge hiding (−0.177) is greater than that of felt obligation (−0.134). The effect value of the difference between the two mediating effects is-0.044, and the 95% confidence interval is [−0.239, 0.168], including 0. This shows that although the mediating effect of psychological safety is greater than felt obligation, there is no significant difference between them.

**Table 4:** Total, direct and indirect effects.

	Effect	BootSE	BootLLCI	BootULCI
Total effect (FT → KH)	−0.312	0.062	−0.392	−0.147
Direct effect (FT → KH)	−0.002	0.109	−0.216	0.215
Indirect effect 1 (FT → PS → KH)	−0.177	0.071	−0.318	−0.042
Indirect effect 2 (FT → FO → KH)	−0.134	0.055	−0.256	−0.044
Indirect effect 1 - Indirect effect 2	−0.044	0.102	−0.239	0.168

FT = Feeling trusted; PS = Psychological safety; FO = Felt obligation; T = Traditionality; KH = Knowledge hiding; Ind1=Indirect effect 1; Ind2=Indirect effect 2.

### Moderating effect test

The results of the moderating effect test are shown in Table 3. The Model 2 shows that the effect of the interaction of feeling trusted and traditionality to psychological safety is not significant after controlling variables such as gender (*β* = −0.086, *p* = 0.061), which indicates that traditionality has no significant moderating effect on the relationship between feeling trusted and psychological safety, and hypothesis 4 is not supported. The Model 4 shows that the effect of the interaction of feeling trusted and traditionality to felt obligation is significant (*β* = 0.131, *p* < 0.05), which indicates that the moderating effect of traditionality on the relationship between feeling trusted and felt obligation is significant, and hypothesis 5 is supported.

**Figure 2:**
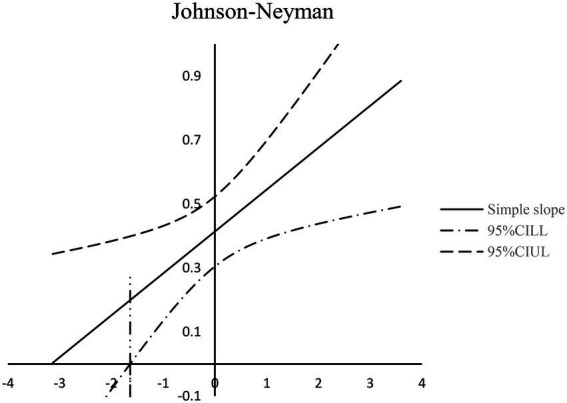
Moderating effect of traditionality between feeling trusted and felt obligation.

In order to show the moderating effect of traditionality more clearly, this study adopted the Johnson-Neyman method for simple slope test (Fang et al., 2015). As shown in Figure 2, when the traditionality (standardized) value range is [−1.638, 3.597] (accounted for 96.14% of the sample size), the confidence interval of the simple slope does not contain 0, and the slope line is above 0. This indicates that feeling trusted significantly positively predicts felt obligation, and the influence of feeling trusted on felt obligation increases with the increase of traditionality. When the traditionality (standardized) value is lower than-1.638, its moderating effect is not significant.

## Discussion

### Research conclusion

Knowledge hiding is indeed a negative behavior which is not conducive to organizational development (Connelly et al., 2012). Based on the questionnaire data, this paper empirically studies the influence of feeling trusted by superiors on nurses’ knowledge hiding, puts forward a parallel mediate model of psychological safety and felt obligation, and investigates the moderating effect of traditionality. Firstly, we found that feeling trusted is negatively related to nurses’ knowledge hiding, which supported hypothesis 1. This result is consistent with the relationship between trust and knowledge hiding in previous research and supports social exchange theory (Liu, 2015; Yao et al., 2020; Erkutlu and Chafra, 2021). The social exchange mechanism shows that one party’s trust has an important influence on the trusted person’s behavior (Sun et al., 2018). When nurses feel trusted by their superiors, they may become the beneficiaries of more practical benefits. In view of this, nurses are more likely to be motivated to make more commitments to this exchange relationship, and may be more willing to fulfill their supervisors’ expectations. However, knowledge hiding behavior is obviously contrary to the expectations of superiors. Therefore, when feeling trusted, the knowledge hiding behavior of nurses will be restrained. That is to say, feeling trusted by superior will help nurses reduce knowledge hiding behavior.

Secondly, feeling trusted can affect nurses’ knowledge hiding through psychological safety, hypothesis 2 is supported. Feeling trusted can significantly enhance nurses’ psychological safety, which is consistent with previous studies (Ma and Wang, 2014; Liu et al., 2018; Song et al., 2020). As a member of the organization, the evaluation of superiors has an important influence on nurses’ psychological safety. Feeling the trust from supervisor makes nurses realize that their role in the organization is recognized, and feel safe and relaxed about the working environment, thereby reducing nurses’ uncertainty and risk perception of exchange relationship, and meeting their psychological safety needs (Liu et al., 2018). Then, high psychological safety facilitates individuals to be more motivated to communicate and share work-related knowledge with others, and reduce knowledge hiding behavior, which is also consistent with previous studies (He et al., 2020). Therefore, feeling trusted by supervisors will meet the psychological safety needs of nurses and thereby inhibit their knowledge hiding behavior.

Thirdly, felt obligation plays a mediate role between feeling trusted and knowledge hiding, which supports hypothesis 3. The trust of superiors brings psychological and resource support to nurses. In order to return the benefits provided by their superiors, nurses will have more sense of obligation to their superiors (organizations), that is, they hold a belief that they should care for their superiors’ well-being and help them achieve their goals based on reciprocal norms. In order to fulfill these obligations, nurses will be motivated to engage in behaviors that meet the expectations of their superiors and reduce harmful behaviors to the organization. Therefore, when feeling trusted, the nurses’ felt obligation increases due to the response to the trust of their superiors and the restriction of reciprocity norms, thus inhibiting knowledge hiding behavior. Previous study has also found that feeling trusted can arouse felt obligation to encourage individuals to engage in beneficial behaviors, such as increasing job participation and reducing turnover intention (Skiba and Wildman, 2019). Therefore, feeling trusted will affect nurses’ knowledge hiding through felt obligation.

Finally, traditionality positively moderates the influence of feeling trusted on felt obligation. Specifically, feeling trusted can significantly increase the felt obligation of high traditional nurses, but it has a weak influence on low traditional nurses, indicating that Hypothesis 5 is supported. Superior is one of the representatives of the authority. Feeling trusted by superiors makes nurses have the willingness and behavior to pay back. For nurses with high traditionality, when they feel trusted by superiors, their awareness of “paying back” are stronger because they obey the authority and are sensitive to grade differences (Zhang, 2017). Therefore, feeling the trust of an authoritative “superior” has a greater impact on high-traditional nurses. For nurses with low traditionality, feeling the trust of their superiors will also arouse their felt obligation. However, because they pursue the principle of fairness and are not sensitive to grade differences, they do not treat the trust of their supervisors with excessive concern and the effect of feeling trusted on them is weak.

However, traditionality has no significant moderating effect on the relationship between feeling trusted and psychological safety, which is inconsistent with hypothesis 4. Although nurses with different levels of traditionality have different concerns and sensitivities to their superiors’ attitudes towards themselves, feeling trusted makes nurses feel psychological safety is no significant difference due to the traditional level. The possible reason is that psychological safety is a subjective feeling of whether one’s environment is safe and reliable. Previous studies have found that its changes are greatly influenced by external factors such as interpersonal relationships, group interaction, leadership and management style, and organizational norms (Kahn, 1990). Traditionality, however, describes the inherent traditional values of individuals, so it may not play a role in the change process of psychological safety. Tang (2018) research also found that the influence of leadership characteristics on psychological safety was not moderated by traditionality, which supported our conclusion.

### Theoretical contribution

There are a few theoretical contributions. Firstly, this study broadens the research perspective of trust, an important predictor of knowledge hiding. Trust occurs in a binary relationship, including the trustor and the trustee. Previous studies mainly focused on the trustor, discussing how the trustor’s trust in others affects his/her knowledge hiding behavior. The current paper explores the influence and mechanism of feeling trusted on knowledge hiding behavior from the perspective of trustee. This expands antecedent variables of knowledge hiding, responds to Brower et al. (2000) call to explore interpersonal trust from the perspective of the trusted person and makes up for the deficiency of previous research on this perspective.

Secondly, based on social exchange theory, this study introduced a dual-path mediation model of psychological safety and felt obligation, and revealed the complex psychological mechanism in the process of social exchange. The research conclusion of this paper shows that, on the one hand, feeling trusted from superiors reduces the uncertainty and risk assessment of exchange relationship perceived by subordinates, and makes subordinates’ psychological safety satisfied, so as to “actively” make behaviors beneficial to superiors and organizations, and establish and maintain exchange relationship with superiors. On the other hand, subordinates’ felt obligation generated from feeling trusted will push subordinates to “have to” or “ought to” do something in return, forming a positive but somewhat “passive” social exchange. Therefore, this paper explains the mediating mechanism of feeling trust affecting knowledge hiding behavior from the perspectives of “active” and “passive,” which is helpful to deepening scholars’ understanding of the potential mechanisms between feeling trusted and knowledge hiding.

Finally, by testing the moderating effect of traditionality, the boundary conditions of feeling trusted are enriched. Existing studies mainly focus on the individual cognitive factors of organizational situation, such as power distance, organizational based self-esteem and norm strength, when discussing the boundary conditions of feeling trusted affecting individual attitudes and behaviors (Wang and Zhang, 2016; Liu et al., 2018; Zheng et al., 2019), the attention to individual characteristic factors is still very limited. This study selects traditionality as the potential boundary condition, and finds that the influence of feeling trusted on felt obligation varies with the level of traditionality. Therefore, the exploration of the moderating role of traditionality is a useful supplement to the boundary conditions of feeling trusted.

### Practical implications

The conclusions of this research have some implications for nursing management practice. First of all, feeling trusted is negatively correlated with knowledge hiding behavior, that is, the higher the degree of feeling trusted, the less knowledge hiding behavior of nurses. Therefore, we suggest that nurses managers should pay attention to the application of trust strategy and improve nurses’ feeling trusted level. Making employees feel the trust from their superiors is the best strategy to give employees responsibility (Lau and Lam, 2008; Salamon and Robinson, 2008). Nurse managers should not only recognize and trust nurses in their hearts, but also convey trust signals to nurses as clearly as possible to ensure that nurses can perceive their trust well. For example, by giving important work to nurses, moderately expanding nurses’ independent decision-making power, strengthening personal contacts and revealing important information at work, nurse managers can express their trust to nurses more explicitly (Liu et al., 2018; Guinot et al., 2021), which is conducive to the establishment of social exchange relationships, so that nurses are willing to reduce knowledge hiding behavior.

Secondly, psychological safety has a negative effect on knowledge hiding, indicating that cultivating and improving nurses’ psychological safety is also an effective way to reduce knowledge hiding. Therefore, we suggest that nurse managers should care about nurses’ psychological state in the organization and help nurses to keep a good psychological safety perception. Nurse managers can improve nurses’ psychological safety by establishing a fair and reasonable organizational system, creating a safe and reliable organizational atmosphere and adopting a constructive leadership style. Besides, the mediating role of psychological safety between feeling trusted and knowledge hiding also suggests that managers can give nurses full trust to meet their psychological safety needs and correspondingly reduce knowledge hiding.

Thirdly, this study confirmed that the stronger nurses’ felt obligation is, the more helpful it is to reduce knowledge hiding behavior. Therefore, how to enhance nurses’ felt obligation level is a question that nursing managers need to think about. Felt obligation is usually a positive belief generated by positive stimulation from superiors or organizations, such as trust, support and recognition (McMillian and Albrecht, 2005; Chen X. et al., 2021). Therefore, as the agent of the organization, the superior should pay attention to giving positive stimulation to the nurses. Managers can enhance their sense of identity, belonging and responsibility to the organization by giving them material and spiritual support, maintaining a high-quality leadership-member exchange relationship with the nurses and establishing a good psychological contract with the nurses (Zhou et al., 2021; Yu J. et al., 2022), so as to achieve the purpose of reducing knowledge hiding behavior.

Finally, nurses’ attention and reaction to their superiors’ trust varies from person to person. Compared with individuals with low traditionality, feeling trusted has a more significant impact on felt obligation of individuals with high traditionality. Therefore, when expressing trust, nurse managers should also consider personal characteristics of subordinates and flexibly choose trust strategies for different individuals, so as to better manage knowledge hiding behaviors in the organization. This requires nurse managers to strengthen communication and contact with their subordinates, and to be familiar with and master the individual traditionality characteristics of nurses. For highly traditional nurses, managers can stimulate their strong sense of obligation to the organization by enhancing their perception of being trusted. For low-traditional nurses, according to the characteristics of low-traditional individuals who emphasis on the principle of fairness, managers can establish a reward mechanism for knowledge sharing and a fair evaluation system to encourage knowledge sharing and reduce knowledge hiding.

## Limitations and future research

First of all, there is a process from feeling trusted to the change of the behavior, and the cross-sectional data cannot reflect the process mechanism of feeling trusted affecting nurses’ knowledge hiding behavior. Future research should consider the order of psychological perception to behavior change and collect longitudinal data in batches to verify causality.

Secondly, the participants in this study are all from China, and traditionality is also a value variable with oriental characteristics. Whether the research results are suitable for other cultural backgrounds needs further verification. People are in a specific cultural background, and their behavior characteristics and decision-making methods may vary with different cultural backgrounds (Kashima et al., 1995). For instance, Boz Semerci (2019) found that in a highly individualistic culture, the negative impact of conflict on knowledge hiding is stronger. Hiller et al. (2006) found that in the context of collectivism culture, members of organizations are more willing to share knowledge actively. Therefore, future research can be carried out in other cultural contexts which may provide more evidence for the relationship between feeling trusted and knowledge hiding. In addition, cross-cultural studies that integrate different cultural backgrounds can often draw more universal conclusions (Segall et al., 1998), so future research can also try cross-cultural research.

Thirdly, based on the social exchange theory, this study found that feeling trusted can weaken the knowledge hiding behavior by enhancing nurses’ psychological safety and felt obligation, which is consistent with many studies focusing on the positive influence of feeling trusted (Lau et al., 2014; Nerstad et al., 2018; Zheng et al., 2019). However, some studies have pointed out that feeling trusted is a double-edged sword, and it also has a dark side (Baer et al., 2015; Wang and Huang, 2019). Baer et al. (2015) argue that perceived trust can also be a stressful experience. Being trusted by superiors means that employees will be given more work and have a reputation that requires effort to maintain. According to conservation of resource theory, feeling trusted will bring workplace anxiety and emotional exhaustion to subordinates (Baer et al., 2015; Wang and Zhang, 2016; Chen H. et al., 2021), which are not conducive to reducing knowledge hiding behavior (Skerlavaj et al., 2018; Guo et al., 2021). Therefore, it is also of great research value to explore whether feeling trusted will have different effects on knowledge hiding through other mechanisms and whether there is a complicated relationship between them.

Lastly, this study mainly explores how to reduce nurses’ subjective knowledge hiding tendency and behavior through organizational behavior management inside the hospital. However, some studies have noticed that digital technology can be applied to knowledge management in the field of healthcare, which contributes to knowledge sharing and improve work efficiency and nursing quality (Hovlid et al., 2012; Ajayi et al., 2019; Abbate et al., 2022).Therefore, how to rely on various digital technologies in the process of digital transformation of medical services to find paths to reduce knowledge hiding behavior in organizations from an objective point of view is also the future research direction.

## Data availability statement

The raw data supporting the conclusions of this article will be made available by the authors, without undue reservation.

## Ethics statement

The studies involving human participants were reviewed and approved by Henan University Key Laboratory of Psychology and Behavior Research Psychology Research Ethics Committee. Written informed consent for participation was not required for this study in accordance with the national legislation and the institutional requirements.

## Author contributions

GL and YL provided the idea, designed this study, and wrote the manuscript. YD, HT, YZ and HH contributed to data collection and data analysis. CC contributed to revised this manuscript. All authors contributed to the article and approved the submitted version.

## Funding

This research was funded by the Research topic of Kaifeng philosophy and social science planning, grant number ZXSKGH-2022-1058, the Key Program of Research and Practice on Undergraduate Teaching Reform of Henan University, grant number HDXJJG2020-25, and the Survey Subject of Henan Federation of Social Sciences Circles: “Research on the Status Quo and Cultivation Mechanism of Social and Emotional Abilities of Youth in Henan Province”, grant number SKL-2022-55.

## Conflict of interest

The authors declare that the research was conducted in the absence of any commercial or financial relationships that could be construed as a potential conflict of interest.

## Publisher’s note

All claims expressed in this article are solely those of the authors and do not necessarily represent those of their affiliated organizations, or those of the publisher, the editors and the reviewers. Any product that may be evaluated in this article, or claim that may be made by its manufacturer, is not guaranteed or endorsed by the publisher.
